# Contribution à l’établissement des plages de référence des immunoglobulines E totales: une étude transversale chez des donneurs de sang à Abidjan (Côte d’Ivoire)

**DOI:** 10.11604/pamj.2025.52.2.48346

**Published:** 2025-09-01

**Authors:** Aya Ursule Aniela Assi, Adjoumanvoulé Honoré Adou, Amah Patricia Victorine Goran-Kouacou, Lasme Roselle Charline Memel, Oppong Richard Yéboah, Brou Doris Yvonne-Fleury Oura, Yida Jocelyne Seri, Angbonon Tychique Elysée Attoukoula, Kouabla Liliane Siransy, Koffi N'guessan, Séry Romuald Dassé

**Affiliations:** 1 Département d'Immunologie-Allergologie, Unité de Formation et de Recherche des Sciences Médicales d'Abidjan, Université Félix Houphouët-Boigny, Abidjan, Côte d'Ivoire,; 2 Laboratoire d'Immunologie et d'Hématologie, Centre Hospitalier Universitaire de Cocody, Abidjan, Côte d'Ivoire,; 3 Département d'Immunologie-Allergologie, Unité de Formation et de Recherche des Sciences Médicales de Bouaké, Université Alassane Ouattara, Bouaké, Côte d'Ivoire,; 4 Centre National de Transfusion Sanguine, Treichville, Abidjan, Côte d'Ivoire

**Keywords:** IgE totales, normes de référence, donneurs de sang, Côte d’Ivoire, Total IgE, reference standards, blood donors, Ivory Coast

## Abstract

**Introduction:**

les immunoglobulines E (IgE) totales sont largement utilisées comme biomarqueurs dans le diagnostic des maladies allergiques. Leur interprétation repose généralement sur des valeurs de référence occidentales, sans tenir compte des spécificités épigénétiques propres aux pays tropicaux. En Afrique subsaharienne, plusieurs facteurs peuvent fortement influencer les niveaux d'IgE. L'objectif de cette étude est d'établir des plages de référence des IgE totales chez des donneurs de sang à Abidjan, considérés comme témoins sains dans notre contexte.

**Méthodes:**

nous avons mené une étude transversale analytique de septembre à novembre 2023 au Centre national de transfusion sanguine d'Abidjan. Un échantillon aléatoire de 136 donneurs de sang volontaires, asymptomatiques, âgés de 18 à 60 ans, a été inclus après obtention d'un consentement éclairé. Chaque participant a bénéficié d'un hémogramme complet, d'un dosage des IgE totales par la méthode ELFA sur automate VIDAS, ainsi que d'un examen parasitologique des selles. L'analyse statistique a été réalisée à l'aide du logiciel SPSS v.29.0, en utilisant les tests t de Student et ANOVA pour la comparaison des moyennes, et le test de corrélation de Pearson pour explorer les relations entre variables quantitatives.

**Résultats:**

l'âge moyen des participants était de 34,5±11,8 ans. Les hommes représentaient 94,9% de l'échantillon. Le taux moyen d'IgE était de 347,1±598,6 KU/L, avec 41,2% des donneurs qui avaient un taux >150 KU/L. Une corrélation négative significative a été observée entre les taux d'IgE et l'âge (r = -0,37; p = 0,03), ainsi qu'une corrélation positive entre les taux d'IgE et le nombre de polynucléaires éosinophiles (r = 0,41; p = 0,014).

**Conclusion:**

cette étude met en évidence des taux moyens d'IgE totales supérieurs aux standards internationaux, probablement en lien avec des spécificités régionales. Ces résultats plaident pour l'établissement de normes locales afin d'adapter leurs interprétations aux réalités ivoiriennes.

## Abstract (en)

**Introduction:**

the total immunoglobulins E (IgE) is used as a biomarker in the diagnosis of allergic diseases. Its interpretation is generally based on reference values established in Western populations, without considering the epigenetic and environmental specificities of tropical countries. In sub-Saharan Africa, factors such as living conditions, polyparasitism, and the diversity of allergenic exposures can strongly influence the IgE levels. The main objective of this study is to establish reference ranges for the total IgE in blood donors in Abidjan as healthy controls in our context.

**Methods:**

we conducted an analytical cross-sectional study from September to November 2023 at the National Blood Transfusion Centre in Abidjan. A random sample of 136 voluntary, asymptomatic blood donors aged 18 to 60 years was included after obtaining informed consent. Each participant underwent a complete blood count, total IgE measurement using the ELFA method on the VIDAS® automated platform, and a parasitological examination of stool samples. Statistical analyses were performed using SPSS version 22.0, with Student's t-test and ANOVA applied for comparing means, and Pearson's correlation test used to assess relationships between quantitative variables.

**Results:**

the mean age of participants was 34.5±11.8 years. Men represented 94.9% of the sample. The mean IgE level was 347.1±598.6 KU/L, with 41.2% of donors having a level >150 KU/L. A significant negative correlation was observed between IgE levels and age (r = -0.37; p = 0.03), as well as a positive correlation between IgE levels and eosinophil count (r = 0.41; p = 0.014).

**Conclusion:**

this study shows average levels of total IgE higher than international standards, probably related to regional specificities. These results support the establishment of local standards to adapt the interpretation to Ivorian realities.

## Introduction

L'IgE est un isotype d'anticorps produit par les lymphocytes B sous l'influence de cytokines de type Th2, principalement l'interleukine-4 (IL-4) et l'interleukine-13 (IL-13), dans le cadre des réponses immunitaires dirigées contre les allergènes et les parasites helminthiques [1,2]. Une fois sécrétée, l'IgE se lie de manière irréversible aux récepteurs FcεRI des mastocytes et des basophiles, déclenchant la libération immédiate de médiateurs inflammatoires lors d'une réexposition à l'antigène [1,3]. Ce mécanisme est central dans la physiopathologie des maladies allergiques telles que la rhinite allergique, l'asthme et la dermatite atopique [3]. Cependant, les taux sériques d'IgE totales sont influencés par de nombreux déterminants biologiques et environnementaux, notamment l'âge, le sexe, l'atopie, le tabagisme, le niveau socio-économique, les expositions chroniques aux allergènes, et surtout, en milieu tropical, la charge parasitaire [4-7]. Dans les pays industrialisés, les valeurs de référence de l'IgE totale sont relativement bien définies et oscillent généralement entre 1,5 et 144 KU/L chez l'adulte sain [8,9]. Toutefois, ces seuils diagnostiques, souvent dérivés de cohortes européennes ou nord-américaines, ne tiennent pas compte des spécificités immuno-épidémiologiques des régions subsahariennes, caractérisées par un polyparasitisme endémique et une exposition antigénique intense dès le jeune âge [6,10]. Dans ces contextes, une hyperimmunoglobulinémie E peut être observée en dehors de toute pathologie allergique manifeste, ce qui rend difficile l'interprétation des résultats d'IgE totales si l'on se base uniquement sur des normes exogènes [6,10,11]. L'absence de seuils locaux adaptés peut ainsi conduire à une surestimation du statut atopique ou, au contraire, à la méconnaissance d'un déséquilibre immunitaire sous-jacent. Or, les données de référence sur les niveaux basaux d'IgE dans les populations africaines, en particulier chez des sujets apparemment sains, demeurent rares [11,12]. En Côte d'Ivoire, où les maladies allergiques constituent un problème de santé publique émergent, l'utilisation de seuils de référence inappropriés complique leur diagnostic et leur prise en charge [5,13]. De plus, l'identification de profils IgE anormalement élevés chez des donneurs de sang pourrait avoir un intérêt en transfusion, afin de limiter le risque de réactions indésirables chez les receveurs. L'établissement de valeurs de référence spécifiques à notre population est donc crucial, tant pour le diagnostic allergologique que pour la sécurité transfusionnelle. Cette étude avait pour objectif de déterminer les taux sériques d'IgE totales chez des donneurs de sang à Abidjan afin de contribuer à une meilleure interprétation des profils immunologiques locaux et à l'amélioration des critères diagnostiques en allergologie dans les régions subsahariennes.

## Méthodes

**Type et cadre de l'étude:** il s'agissait d'une étude transversale analytique menée sur une période de trois mois, de septembre à novembre 2023. Le recrutement des participants s'est fait au Centre national de transfusion sanguine (CNTS) d'Abidjan. Les analyses biologiques ont été effectuées dans le laboratoire d'Immunologie et d'Hématologie du centre hospitalier universitaire (CHU) de Cocody et à l'Institut Pasteur de Côte d'Ivoire, deux structures reconnues pour la qualité et la fiabilité de leurs examens spécialisés.

**Population d'étude, critères d'inclusion et d'exclusion:** la population d'étude était constituée de donneurs volontaires de sang, âgés de 18 à 60 ans, recrutés au CNTS d'Abidjan. Les critères d'inclusion étaient les suivants: i) être en bonne santé apparente selon les critères de sélection transfusionnelle; ii) avoir donné un consentement éclairé écrit. Les critères d'exclusion ont exclus les participants présentant au moins un des critères suivants: i) antécédents d'atopie (rhinite allergique, eczéma, asthme allergique, etc.); ii) allergies sévères connues; iii) tabagisme actif; iv) consommation régulière d'alcool (plus de deux consommations par semaine); v) antécédents d'infestation parasitaire récente (moins de 3 mois); vi) exposition professionnelle ou domestique à des allergènes connus (poussière, moisissures, animaux, produits chimiques inhalés); vii) échantillon biologiquement ou techniquement non exploitable (volume insuffisant, hémolyse, conservation inadéquate, ou délai excessif de traitement).

Ces conditions, bien que compatibles avec le don de sang dans certains cas, sont reconnues pour modifier significativement les taux sériques d'immunoglobulines E. Leur exclusion visait à limiter les biais de confusion et à garantir la représentativité des valeurs de référence dans une population témoin saine.

**Echantillonnage et taille de l'échantillon:** la taille minimale d'échantillon a été estimée à l'aide de la formule de Schwartz pour l'estimation d'une proportion dans une population infinie.


$$n=z^{2}\times p\left(1-p\right)/d^{2}$$

n = taille de l'échantillon; Z = 1,96 (valeur critique pour un intervalle de confiance à 95%); p = 0,5 (proportion attendue, choisie par prudence en l'absence de données locales); d = marge d'erreur tolérée (0,085). En appliquant cette formule, la taille minimale requise était de 133 participants. Après ajustement pour un taux de non-réponse éventuel de 3%, nous avons retenu un effectif final de 136 donneurs, afin de garantir une puissance statistique suffisante.

**Collecte des données:** les données ont été recueillies à l'aide d'un questionnaire standardisé comportant des informations démographiques (âge, sexe) et médicales (antécédents allergiques, pathologies chroniques, prise de médicaments, tabagisme, alcoolisme).

Les prélèvements sanguins ont été réalisés dans des tubes *ethylenediaminetetraacetic acid* (EDTA) pour l'hémogramme et des tubes secs pour le dosage des IgE totales. Un récipient propre et hermétiquement fermé a été remis à chaque participant pour le recueil des selles, destinées à l'examen parasitologique. Les échantillons ont été analysés dans un délai de 3 heures après collecte, avec triple emballage selon les normes en vigueur. Pour les sujets préférant effectuer le recueil à domicile, des consignes écrites et orales, expliquant les conditions de prélèvement et d'acheminement, ont été fournies.

Analyses biologiques: i) l'hémogramme a été réalisé sur un automate Sysmex Xn 550, utilisant les principes combinés d'impédancemétrie, de cytométrie en flux et de spectrophotométrie; ii) le dosage des IgE totales a été effectué par méthode *Enzyme-Linked Fluorescent Assay* (ELFA) sur automate VIDAS (référence IVD7006414), reposant sur une méthode immunoenzymatique en sandwich avec détection finale par fluorescence. Une concentration sérique <150 kU/L était considérée comme normale, conformément aux seuils de référence du laboratoire; iii) l'examen parasitologique des selles, réalisé chez tous les participants, associait une observation microscopique directe à l'état frais et une concentration selon la méthode de Ritchie modifiée. Cette double approche a permis de rechercher systématiquement les principales formes parasitaires (œufs, larves, kystes, trophozoïtes). Elle visait à détecter des infestations intestinales asymptomatiques, en particulier à helminthes, dont la stimulation immunitaire de type Th2 peut induire une élévation des taux d'IgE totales.

**Variables étudiées:** les principales variables dépendantes étaient les taux sériques d'IgE totales. Les variables indépendantes incluaient l'âge, le sexe, les résultats de l'hémogramme (notamment les polynucléaires éosinophiles) et la présence de parasitose. Les variables quantitatives ont été analysées en tant que telles, sans catégorisation a priori.

**Analyse statistique:** les données ont été analysées à l'aide du logiciel SPSS version 29.0. Les variables quantitatives ont été exprimées sous forme de moyennes avec leurs écarts-types. Les comparaisons de moyennes entre groupes ont été effectuées à l'aide des tests t de Student et d'ANOVA. Les corrélations entre les taux d'IgE totales et les variables continues telles que l'âge ou le nombre de polynucléaires éosinophiles ont été examinées à l'aide du coefficient de corrélation de Pearson. Le seuil de signification statistique a été fixé à p < 0,05. Les données manquantes ont été systématiquement contrôlées et, le cas échéant, exclues des analyses sans recours à une méthode d'imputation.

**Considérations éthiques:** cette étude a obtenu l'approbation du comité éthique du Centre national de transfusion sanguine. Le consentement éclairé écrit a été obligatoire pour tous les participants. Les informations collectées ont été anonymisées par l'attribution d'un identifiant alphanumérique. Les données ont été traitées de manière confidentielle, et les participants ont été informés de leur droit de retrait à tout moment sans justification.

## Résultats

**Figure 1:**
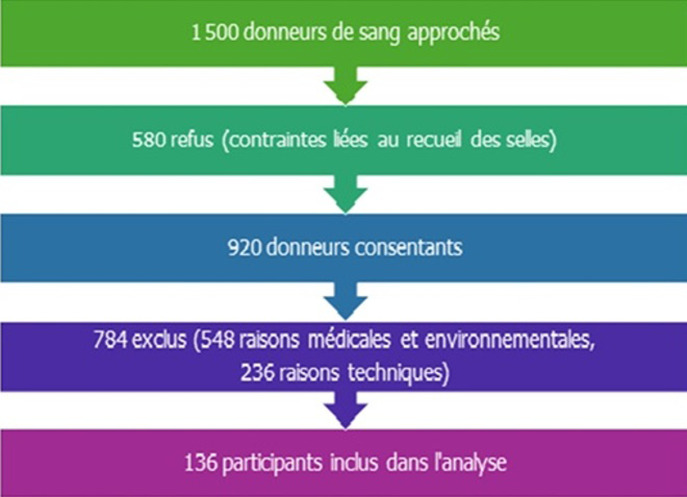
diagramme de flux des participants inclus dans l'étude

**Participants et sélection:** sur les 1500 donneurs de sang ciblés pendant la période d'étude, 580 ont refusé de participer, en grande partie en raison du recueil des selles. Parmi les 920 donneurs consentants, 548 ont été exclus pour des critères médicaux, et 236 pour des raisons techniques (notamment liées au délai d'acheminement ou à la qualité des échantillons de selles). Au total, 136 participants remplissant tous les critères ont été inclus dans l'analyse (Figure 1).

**Caractéristiques épidémiologiques et biologiques de la population:** l'étude a porté sur 136 donneurs de sang. La population était majoritairement masculine (94,9%), avec un sex-ratio de 16. L'âge moyen des participants était de 34,5±11,8 ans, avec des extrêmes de 19 et 65 ans. La majorité des sujets (79,4%) appartenaient à la tranche d'âge de 25 à 65 ans (Tableau 1).

**Tableau 1:** répartition des donneurs de sang selon l'âge, le sexe et les taux d'IgE totales

Variables	Catégories	Effectif (n)	Pourcentage (%)
**Tranches d'âge (ans)**	[18 - 24]	28	20,6 %
	[25 - 65]	108	79,4 %
	Âge moyen ± ET	34,5 ± 11,8	-
	Extrêmes	19 - 65	-
**Sexe**	Féminin	8	5,9 %
	Masculin	128	94,1 %
	Sexe-ratio (H/F)	16	-
**IgE totales (kU/L)**	< 150	80	58,8 %
	> 150	56	41,2 %
	Moyenne ± ET	347,1 ± 598,6	-
	Extrêmes	0,84 - 2549,2	-

La concentration moyenne des IgE totales était de 347,1±598,6 kU/L, avec des valeurs extrêmes comprises entre 0,84 et 2549,16 kU/L. Un taux élevé d'IgE (>150 kU/L) a été observé chez 41,2% des donneurs (n = 56) (Tableau 1). Le taux moyen de polynucléaires éosinophiles était de 149,12±137,52 éléments/mm3. L'examen parasitologique des selles a permis d'identifier des helminthes de type Ascaris lumbricoides chez 4 donneurs (2,9%).

**Figure 2:**
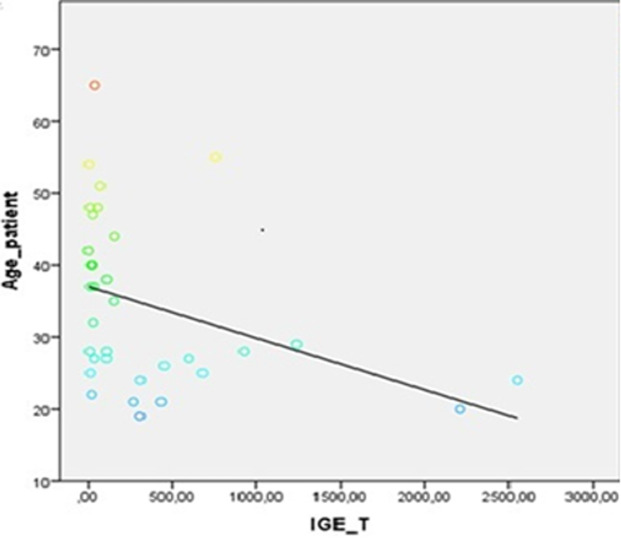
corrélation entre les taux sériques d'IgE totales et l'âge des donneurs

**Figure 3:**
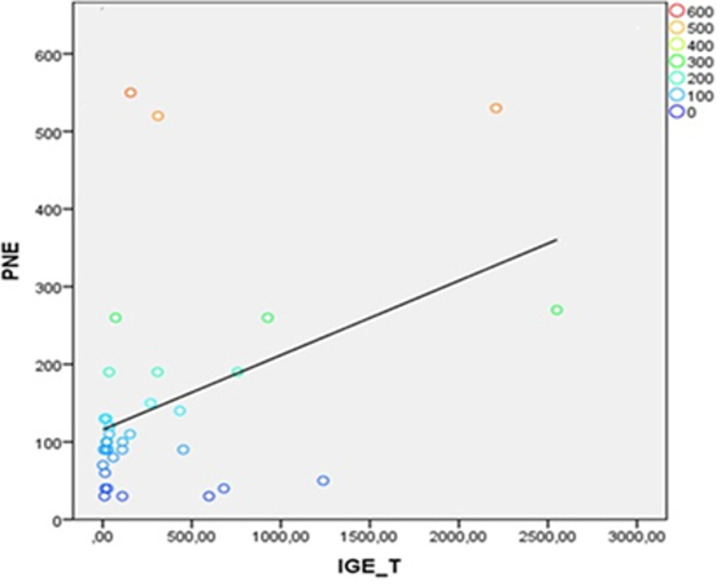
corrélation entre les taux sériques d'IgE totales et les taux absolus de polynucléaires éosinophiles

**Corrélations entre les IgE totales et les variables biologiques:** une corrélation négative significative a été observée entre l'âge des participants et les taux d'IgE totales (r = -0,37; p = 0,03), traduisant une tendance à la baisse des IgE avec l'augmentation de l'âge (Figure 2). En parallèle, une corrélation positive significative a été mise en évidence entre les IgE totales et le taux absolu de polynucléaires éosinophiles (r = 0,41; p = 0,01), suggérant une association entre l'éosinophilie et l'élévation des IgE (Figure 3).

## Discussion

L'objectif principal de cette étude était de déterminer les valeurs de référence du taux sérique des IgE totales dans une population ivoirienne saine, représentée ici par des donneurs de sang. La concentration moyenne mesurée était de 347,1±598,6 kU/L, avec 41,2% des participants présentant un taux supérieur à 150 kU/L. Ce niveau dépasse largement les valeurs de référence classiquement rapportées dans les populations non atopiques européennes ou sud-américaines, comme celle de Spalding *et al*. qui rapportait une moyenne de 54,4 kU/L chez 86 sujets sains non atopiques à Porto Alegre [14].

Malgré l'exclusion rigoureuse des donneurs présentant des antécédents atopiques ou des facteurs confondants déclarés, l'élévation marquée des IgE pourrait s'expliquer par une hypersensibilité environnementale diffuse ou un état de polysensibilisation sous-jacent, fréquent en milieu tropical [13]. Une autre hypothèse plausible serait la persistance d'une stimulation antigénique de bas grade, potentiellement liée à des parasitoses infra-cliniques. Cependant, l'examen parasitologique des selles n'a mis en évidence des helminthes que chez 2,9% des participants. Cette faible prévalence contraste avec le contexte endémique et peut s'expliquer par la sensibilité limitée de l'examen parasitologique standard, la qualité hétérogène du recueil à domicile et l'absence de techniques moléculaires plus sensibles.

Les taux élevés d'IgE pourraient également refléter des déséquilibres immunitaires non allergiques. Liphaus *et al*. ont observé des taux moyens d'IgE atteignant 271,6 kU/L chez des patients atteints de lupus juvénile sans allergie ni parasitose, suggérant un rôle de l'activation polyclonale ou d'une dérégulation de l'axe Th2 dans certaines pathologies auto-immunes [15]. L'analyse des facteurs influençant les IgE a mis en évidence une corrélation négative significative avec l'âge (r = -0,37; p = 0,03), traduisant une décroissance progressive des taux au fil des décennies. Ce phénomène est bien documenté dans la littérature [5,16] et s'expliquerait par la maturation du système immunitaire, la réduction des expositions antigéniques précoces, et la sénescence immunitaire [16].

Notre observation corrobore les données de Fadil *et al*., selon lesquelles les taux d'IgE augmentent durant l'enfance et l'adolescence, en lien avec une exposition plus fréquente aux allergènes et aux agents parasitaires, avant de diminuer à l'âge adulte [16]. Ce profil évolutif suggère que les valeurs de référence devraient idéalement être stratifiées selon les tranches d'âge. En parallèle, une corrélation positive avec les polynucléaires éosinophiles (r = 0,41; p = 0,01) a été observée, renforçant l'idée d'une activation immunitaire de type Th2. Cette association a été décrite par Girard *et al*., notamment dans le cadre des helminthiases, où l'éosinophilie s'accompagne souvent d'une élévation des IgE totales [17]. Toutefois, Chabasse *et al*. n'ont pas observé de relation similaire chez des patients souffrant de pathologies allergiques, ce qui suggère que cette corrélation pourrait dépendre du contexte étiologique: allergique ou parasitaire [18]. Ces données soulignent l'importance de prendre en compte le terrain immunologique sous-jacent dans l'interprétation des taux d'IgE. Aucune association significative n'a été observée avec le sexe, ce qui est cohérent avec certaines séries, mais diverge de celles de Levesque qui, chez des adolescents québécois atopiques, a rapporté des taux plus élevés chez les garçons (51,8 KU/L) que chez les filles (38,0 KU/L) [19]. Cette disparité pourrait être liée à des différences d'âge, d'exposition ou de susceptibilité hormonale. Dans l'ensemble, ces résultats soutiennent l'idée que les valeurs normales d'IgE totales sont fortement influencées par les conditions environnementales, infectieuses et immunologiques locales, et que les seuils issus de populations occidentales ne sont pas directement transposables aux contextes tropicaux.

**Limites de l'étude:** la méthodologie transversale ne permet pas d'établir une relation causale entre les taux d'IgE et les facteurs associés. Ensuite, la qualité du recueil des selles, majoritairement effectué à domicile, a pu altérer la détection des parasitoses, en dépit de l'usage de la méthode de concentration. Par ailleurs, certains facteurs potentiellement confondants, tels que l'environnement domestique, les niveaux d'exposition aux allergènes ou les marqueurs immunologiques plus spécifiques (IgE spécifiques, cytokines Th2), n'ont pas été explorés. Enfin, la taille de l'échantillon, bien qu'adéquate pour une estimation descriptive, reste insuffisante pour une modélisation multivariée robuste.

## Conclusion

Les résultats de cette étude rappellent combien il est important d'adapter les repères biologiques aux réalités locales. Dans un contexte tropical comme celui de la Côte d'Ivoire, de nombreux facteurs, souvent silencieux ou non déclarés, peuvent influencer les taux d'IgE totales, sans pour autant relever d'une allergie avérée. Cette complexité souligne les limites d'une application systématique des seuils de référence établis à partir de populations occidentales, qui ne reflètent pas les réalités immuno-environnementales propres à notre contexte. Une meilleure compréhension des profils IgE passe par une approche globale, combinant le dosage des IgE spécifiques, une exploration parasitologique approfondie et une analyse des facteurs environnementaux.

### Etat des connaissances sur le sujet

- Les IgE totales sont classiquement associées aux pathologies allergiques, mais leur élévation peut aussi survenir en contexte parasitaire ou auto-immun, sans lien direct avec un terrain atopique;
- Les seuils de normalité internationalement admis (<150 kU/L) sont établis à partir de populations européennes ou asiatiques, peu représentatives des conditions environnementales des pays tropicaux.

### Contribution de notre étude à la connaissance

- Dans un contexte ivoirien marqué par le polyparasitisme et la sensibilisation environnementale diffuse, près de la moitié des donneurs sains dépassent ce seuil, suggérant que les normes internationales pourraient être inadaptées;
- Nos résultats révèlent chez des sujets considérés sains un taux moyen d'IgE totale supérieur à la norme internationale, d'où l'intérêt d'élaborer des valeurs de référence locales pour une interprétation plus pertinente en pratique diagnostique.

## Remerciements

Nous exprimons notre profonde gratitude à l'ensemble des équipes des laboratoires d'Immunologie et d'Hématologie du CHU de Cocody, ainsi qu'à l'Institut Pasteur de Côte d'Ivoire, pour leur appui technique et logistique tout au long des analyses biologiques. Nous remercions chaleureusement tous les donneurs de sang qui ont accepté de participer à cette étude, pour leur disponibilité et leur précieuse collaboration.
